# Becoming a new parent during the pandemic: experiences of pregnancy, birth, and the postnatal period

**DOI:** 10.1186/s12884-024-07110-w

**Published:** 2025-01-17

**Authors:** Emma Blakey, Daniel Kuria, Michelle McGillion, Fiona Scott

**Affiliations:** 1 School of Psychology, University of Sheffield, Sheffield, UK; 2 School of Education, University of Sheffield, Sheffield, UK; 3 Department of Psychology, University of Warwick, Warwick, UK

**Keywords:** Covid-19, New parenthood, Motherhood, Pregnancy, Birth, Postnatal period

## Abstract

New parenthood in ordinary times can be a vulnerable and unpredictable time. The Covid-19 pandemic brought additional, unprecedented changes to policy and practice that drastically impacted on the experiences of parents. This study aimed to enhance our understanding of the experiences of new parents during the pandemic by qualitatively analysing their experiences. New parents from the UK (*N* = 303; female = 296; male = 7) responded to a survey conducted between 2021–2022 asking about experiences of pregnancy, birth and the postnatal period. Responses were analysed thematically, taking an interpretivist approach, and drawing across the three time periods. Parents reported conflicting feelings, negative feelings and silver linings cutting across eight themes, including: impacts on well-being, feeling without a village, changes to healthcare, atypical social experiences as a new parent, differential impacts on financial and working lives, conflicting feelings around digital technology, anger and worry regarding contradictory government guidance and recommendations for other parents. The findings offer much needed insights into the experiences of new parents during this time and provide some context to the documented elevated levels of perinatal mental health difficulties in new parents during the pandemic. We suggest key recommendations going forwards in the care of new parents now, and in times of future national crisis.

**Supplementary Information:**

The online version contains supplementary material available at 10.1186/s12884-024-07110-w.

## Introduction

The Covid-19 pandemic brought drastic and unprecedented changes to policies and practices that restricted normal liberties and services for all. As governments sought to limit the spread of Covid-19, changes to policy and practice were made that impacted experiences of pregnancy, birth and the postnatal period. These changes cut across health care provision, pre- and postnatal- support, and birth policies; but also working practices and social contact. After comparing the pandemic responses of maternity services around Europe, Lalor et al., [23] concluded that many of these changes were introduced without an evidence base and could be considered antithetical to human rights. We aimed to understand the impact these changes had on new parents, as new parenthood is considered a vulnerable time in ordinary times [39]. Understanding the experiences of new parents during this window of time is critical for two reasons. Firstly, to better understand how vulnerability in systems, disrupted healthcare and changes in policy shapes new parenthood. This may help us to learn lessons for what services and supports need to be preserved in times of future national crises to protect new parents. This is especially important in the context of an increasingly stretched and underfunded maternity healthcare system, wherein reductions to provision have, and may continue to, occur outside of national crises. Secondly, to understand how limited social support impacted the lived experience of new parents. Social support captures a person’s perception of the availability of others to provide emotional, psychological and material resources [28]. Looking at this during this window of time in which it was drastically reduced may illuminate its role in new parenthood.

New parenthood in non-crisis times is a huge life transition characterised by psychological and social adjustment [26]. Changes occurring in biology and psychology are accompanied with rapid and significant changes in identity and social networks, prompting some to characterise new parenthood as a distinct life stage (see [30] for a review). It is therefore not surprising then that new parenthood is considered a vulnerable period for the onset and exacerbation of mental health issues [38]. In the context of the Covid-19 global pandemic, established practices and access to services throughout pregnancy, birth and during the postnatal period changed drastically from standard care [19]. These changes were radical and untested, and while they remained consistent during the first part of the pandemic, in the second half rules started to relax, and practices became inconsistent across areas []. Pre-pandemic data shows that around 20% of women are affected by maternal mental health conditions and these are the leading cause of maternal death in the first year postpartum [29]. Indeed, in 2020, mothers were three times more likely to die by suicide during or up to six weeks postpartum compared to 2017–2019 [25]. Quantitative research has demonstrated elevated anxiety and depression in pandemic-era parents compared to pre-pandemic levels (e.g., [10, 18]). Large surveys documenting elevated maternal mental health issues are valuable, but importantly, they lack the rich contextual information needed to explain *why* this is happening. Without more fine-grained analyses, it is hard to fully understand the negative impact that specific policies and reduced healthcare provision have had on new parents’ well-being and to effectively advocate for targeted support for new parents. We take a holistic view of well-being to consider physical and emotional health, as well as financial health and social interactions with both family and the wider community (see [14]).

**Fig. 1:**
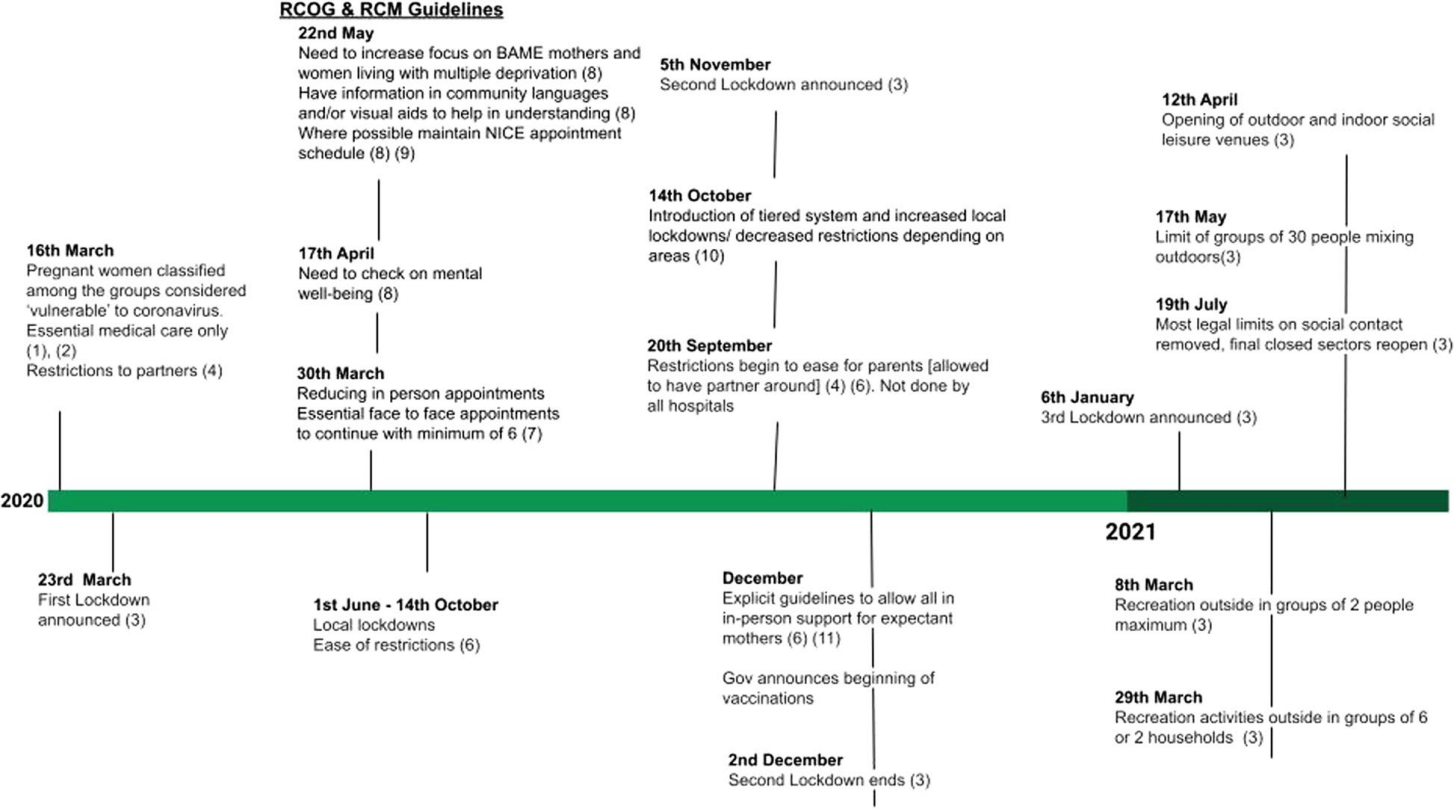
Timeline of key policy changes in the UK during the Covid-19 pandemic

Overarching key policy changes that occurred during the pandemic in the UK can be seen in Fig. 1 and their impacts on parents' access to healthcare and social support between March 2020 and 2021 are briefly summarised here. Policy changes, and communications about policy changes, in the period can be characterised by a lack of clarity, internal contradictions and rapid change. Policy changes also carried very different implications for different groups of pregnant women. In a Press Conference on 16th March 2020, pregnant women were officially categorised as ‘vulnerable’ and advised that it was particularly important for them to avoid all unnecessary social contact [41], though the precise meaning of this was unclear and no central guidance on how this should be operationalised was provided. Pregnant women, but only those who were deemed to be clinically vulnerable, were, much later, advised to shield [40] and structural support mechanisms for these groups put in place. As the UK’s maternity rights charity, Maternity Action, has previously emphasised (2021), women were left in a position of uncertainty about which version of official guidance to follow and what the evidentiary basis for the fluctuating guidance was. The UK’s Chief Medical Officer emphasised that the UK Government were acting 'as a precautionary measure because [they were] early in [their] understanding of [the] virus’, whilst various trusted advisory bodies, including the Royal College of Midwives and Royal College of Obstetricians and Gynaecologists reassured pregnant women that ‘no previous coronavirus has shown evidence of foetal abnormalities and miscarriages’ (RCOG, 2020; RCM, 2020). However, existing work had already evidenced that previous coronaviruses, including SARS and MERS could indeed cause severe adverse pregnancy outcomes, including miscarriage, premature delivery, intrauterine growth restriction and maternal death [11]. This resulted in a range of very different experiences for pregnant women, with some being wrongly told to work in unsafe working conditions and others suffering substantial financial loss when taking steps to avoid these risks [24]. Meanwhile, changes to standard care included a reduction in face-to-face appointments and a move towards telehealth and remote antenatal and postnatal appointments (RCOG, 2020). Specifically, during pregnancy and birth, many parents were prevented from attending prenatal scans, some mothers gave birth alone or birth partners were only allowed to be present for the ‘active labour’ portion of the birth and for a short time afterwards [16, 33]. Reduced staffing due to midwives and other healthcare professionals being redeployed across the sector [12], meant that parents were reportedly less attended to by midwives during their labour and also cared for by a greater number of different midwives than was usual pre-pandemic (Harrison et al., 2021). Many babies were also separated from their mothers after the birth, undoing years of work on the importance of physical contact between mothers and babies for both infant health and the mother’s wellbeing [39]. During the postnatal period, health visiting appointments and parent health checks were limited or changed their usual provision,access to weighing in clinics, feeding support or mental health support were reduced. Due to limits on social contact, many parents were left without the social support they may have experienced through extended family and friends and through meeting other parents. Restrictions on maternity wards remained stringent for the first part of the pandemic and as more became known about the virus midway through 2020, rules relaxed and diversified but large geographical variation in rules remained. As we write this in 2023, healthcare and support for new parents is still not back to pre-pandemic levels. In the UK, maternity and healthcare services remain underfunded, and there are still restrictions on face-to-face appointments and reduced support for feeding and mental health. Many health visitors were redirected to other areas during the pandemic and not at all returned [44].

Emerging qualitative research has started to shed light on the diversity and profundity of parents’ experiences during the pandemic. These studies have demonstrated in particular the negative impacts of changes in practice on well-being during pregnancy [27], levels of trauma during birth [3] and perinatal distress [21]. An important recent study was able to cut across all maternity-related time periods to examine experience of service provision specifically, using a large dataset of mothers who had completed the National Maternity Survey [28]. They reported key themes of ‘chaos’, ‘abandonment’ and ‘aloneness’ in mothers’ experiences of the antenatal and postpartum periods. Another cross-cutting theme suggested that participants particularly valued good care and communication when it happened. McLeish and colleagues concluded that valuable lessons can be learned by understanding the experiences of ‘pandemic parents’ for maternity services going forward, and in particular, that social support may be protective for mothers’ well-being.

The overall objectives of our study were to (a) provide pandemic new parents with an opportunity to share and reflect on their experiences and (b) further understand the impact the pandemic had across multiple areas (e.g., healthcare provision, social interaction, changes to work and finances) on the whole experience of becoming a new parent. From this, we hoped to learn lessons for how we can support new parents going forward, both in potential future crises and in times of non-crisis. Specifically, we were interested in the impacts changes in policy and practice had on new parent well-being, feelings of social support, and experiences of digital technology throughout the whole period to becoming a new parent. We expected to see a negative influence on parent well-being to align with the emerging quantitative data (e.g., [18]). We also expected to see social support come out as important in participants' experiences, particularly as it plays a vital role in buffering stress [28]. Finally, we expected to see diverse experiences on the role of digital technology as many areas of in person provision and support switched to online models, and as a way to connect with wider social networks. While quantitative research has suggested elevated mental health issues in parents during the pandemic, and qualitative research has begun shed light on key themes during particular time periods (e.g., pregnancy during the pandemic) we sought to use a rich qualitative approach to understand experiences across the whole journey on becoming a *new* parent. It is only in looking at all three pivotal time points (pregnancy, birth and postnatal) we can obtain a more holistic picture of the journey to new parenthood and capture changes and complexities in experiences over time. The study had four research questions:

1. How did new parents feel their well-being was impacted?
2. How was new parents’ social support affected?
3. What role did digital technology play in experiences of becoming a parent for the first time during the pandemic?
4. What concerns did parents have for their child’s development during the pandemic?

The aim of the present article was to address all four research questions to present an overall perspective on new parents' experiences, rather than exploring a number of issues in more depth. Whilst we would like to explore the issues in more depth in future work, we consider it a priority to share the headline findings of the study, given the empirical importance of the topic. In doing so, we hope to contribute to discussion on how the pandemic affected the experiences of first time new parents in a way that can contribute to policy and practice supportive of the well-being of first time new parents in both non-pandemic times and in times of possible future crisis.

## Method

### Study design and theoretical approach

We sought to obtain a large sample, to capture the considerable variation that has become apparent in parents’ experiences. To obtain a large sample, we made use of online surveys as a qualitative tool (see [5]), and invited parents to complete a survey with open-ended prompts to share their experiences.

The research design was guided by the overall purpose of the study and the four research questions, that is, a medium-scale qualitative design for exploratory work to build understanding. The theoretical approach was broadly what has been described as interpretative or interpretivist [1]). Specifically, it was guided by the notion that reality is situationally co-constructed and that any qualitative work is to some extent shaped and situated by the research team. The study employed a single method: a qualitative new parent survey. The Covid-19 pandemic forced qualitative researchers to adapt methods, often shifting to online approaches due to legal, practical, and ethical constraints [29, 34]. Our choice of open-ended survey was responsive to the challenges of studying a sensitive topic with potentially vulnerable groups, such as first-time parents, during a global pandemic. This method balanced the empirical need for qualitative insights with ethical considerations, minimising participants' time and organisational demands. While richer data might have come from interviews, the survey allowed us to gather a large sample and it allowed for flexible, detailed responses without disrupting participants' often tight schedules as new parents and without the risks that can from any in-person study designs, a crucial ethical consideration.

### Participants

Participants were invited to complete a Google Form online survey if they became a parent, to an infant under the age of 1 for the first time between September 2019 and July 2022. The invitation to take part in the research was initially shared through Facebook community parent/family groups, and through posters in family community areas. Later, the survey was shared more widely with the National Childbirth Trust through their social media accounts and parent newsletters to increase response rates and through local news articles, radio stations and a TV news channel. Initially, 327 participants took part in the study. Of these, we excluded participants if they indicated they were not a first-time parent (*N* = 13), were outside of the UK (*N* = 10), or had incomplete responses (*N* = 1). This left 303 participants (296 females and 7 males; UK (area not specified) = 135; England = 147, Scotland = 10, Wales = 6, Northern Ireland = 5). See Table 1 for further demographic information on the sample. In recognition of the fact that participants included in research studies are often overwhelmingly ‘WEIRD’ [17] and that NCT membership carries a fee (albeit with discounts for low-income families), we made significant efforts to circulate information about the study to platforms representative of a more diverse parent population, including Facebook community parent/family groups, and through posters in family community areas and supermarkets. Notwithstanding this, the participant cohort was not as diverse as we had hoped in terms of parent ethnicity, self-described gender or parental status (i.e., biological, foster or adoptive parents). We decided not to include a measure of social class due to the sensitive nature of asking about family financial circumstances in a research project that already focused on a sensitive topic.

**Table 1:** Participants’ (*N* = 303) demographic information in terms of ethnicity and the year and month of their babies arrival

**Demographic variable**	N
**Ethnicity**	
White – British White—Another background Mixed ethnicity Asian—Another background Asian – Indian Another background Black – African Black – British Not specified	268 24 5 1 1 1 1 1 1
**Year of birth or arrival of baby**	
2019 2020 2021 2022 Not specified	10 209 78 5 1
**Parent disability**	
No Yes Missing	255 13 35
**Month of birth or arrival of baby**	
**2019**	
December November September October July	3 2 2 2 1
**2020**	
January February March April May June July August September October November December Not specified	9 6 16 25 26 21 16 14 20 21 18 15 2
**2021**	
January February March April May June July August September October November December Not specified	12 12 6 6 7 9 7 5 6 1 2 3 2
**2022**	
January July	4 1

#### Data generation

The qualitative new parent survey was drafted and reviewed in line with the study’s aims and broadly interpretative theoretical approach. We were particularly reflexive as a team at this stage, meeting often to share our own experiences of pandemic new parenthood and discussing practical strategies for ensuring our own experiences, which were themselves diverse, did not overtly influence or constrain the responses our participants were able to give. We wanted to give new parents the opportunity to write as much or as little about their experience as they wanted and to offer relatively open prompts for reflection that were as little ‘leading’ as possible, to give participants the opportunity to answer questions in different ways. It consisted of both open-ended questions and closed-ended questions. In the open-ended questions, we took a qualitative approach and using broad prompts asked participants to share their experiences of pregnancy (or before the baby arrived), birth (or arrival of the baby) and after the baby arrived and the role digital technology played throughout. Our phrasing (‘what stands out for you about the experience?’) was intended to provide parents with opportunities to share reflections on their experiences, without pre-framing these ourselves as either positive or negative. We specifically included terminology in the prompts that would be inclusive of adoptive or foster parents, although responses suggest that only biological parents responded. We also asked the parents what they thought would be helpful for other new families who might go through a similar experience as them. The study's design allowed participants to respond in as much detail as they preferred, aligning with ethical principles for sensitive topics. All but two participants provided responses of at least a few sentences to each question, and some accounts provided a lot of detail. The mean word count was 278 words for each of the three time periods (pregnancy, birth, postnatal). The closed-ended questions gathered demographic information, such as self-identified gender, geographical location, month and year their baby was born or arrived. In the interests of methodological transparency and dependability, relevant sections of the study’s qualitative new parent survey are included in Appendix 1. The study received ethical approval from the University ethics committee and all participants gave their informed consent to take part. The survey took place over a period of ten months, between September 2021 and July 2022.

#### Data analysis and presentation of results

**Fig. 2:**
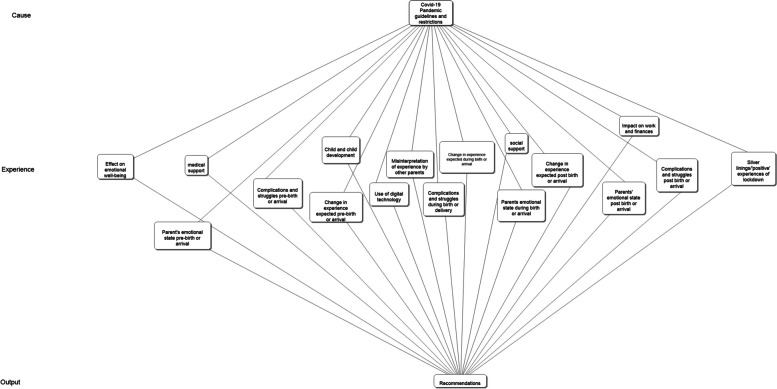
Regrouping of overarching codes

Data analysis adopted a qualitative approach following Braun [7] and Braun and Clarke’s [6] thematic analysis (TA). The responses were imported from the Google Form in a spreadsheet format into NVivo 12 and the open-ended questions were coded in a predominantly inductive manner. This involved an in-depth reading and rereading of the responses by one of the researchers to develop initial codes. The researcher who took a lead on the coding was, however, familiar with some relevant research literature and, as such, the coding was broadly inductive, in the context of some awareness of existing themes and theories. Following Braun's [7] model, the researcher who took the lead in the coding presented a list of codes and excerpts from the participants (to provide exemplification) to the whole research team, alongside a set of potential themes. These codes, excerpts and themes were then discussed and reviewed. At this stage, and in line with the subjective affordances of TA [6], the codes, excerpts and potential themes presented led to a reflexive discussion amongst the research team. It was decided that the proposed themes had not fully captured the diverse experiences of parenting during the pandemic suggested by the fuller list of codes and excerpts. This led to collaborative reviewing and regrouping of the codes with a second researcher, which resulted in the final development of an overarching grouping of themes within the categories ‘cause’, ‘experience’ and ‘output’ (Fig. 2). Significant reviewing and mapping of the codes to the overarching themes and sub-themes finally resulted in a set of themes and sub-themes that the whole research team felt reflected the diversity of the data set. These were named and defined by assigning each theme and sub-theme a descriptive title (Table 2). Eight overarching themes were developed with 2–5 sub-themes per theme. See Table 2 for a list of the themes and sub-themes. Overall, the collaborative nature of the coding and analysis process offered opportunities for the research team to foreground, discuss and resolve particular biases associated in particular with their own identities (in three of four cases) as pandemic new parents themselves. The inclusion of a fourth researcher, who was responsible for the initial coding and who was not himself a pandemic new parent, offered an important alternative perspective on the data.

**Table 2:** Summary table of the themes and sub-themes

Theme	Sub-theme
**1. New parents’ well-being was impacted**	• Feelings of fear and uncertainty • Feelings of loneliness and isolation • Feeling like a failure • Silver linings: feelings of pride • Grappling with internal emotional dissonance (feeling more than one emotion at the same time, e.g. grief/sadness and gratitude/relief/joy)
**2. New parents felt without the expected ‘village’ but some expressed appreciation for small ‘silver linings’**	• Struggling due to lack of practical and emotional social support (before, during and after baby’s arrival) • Grief at missing out on expected key communal milestones. • Silver linings: time for preparing for baby and recovery and bonding as a family • Pandemic experiences were diverse and assumptions, conflicting opinions, comparisons and misinterpretations of experiences exacerbated negative social experiences between parents and their peers and families • Partners struggled with a range of experiences and feelings as a result of being excluded from aspects of the experience (pre, during and post baby arrival)
**3. Changes to healthcare and related support before, during and after baby arrival impacted new parents’ experiences and well-being**	• Pre-and post-natal support was reduced and often very different to standard care • Anxiety around being in hospital (e.g. catching the virus) • Inadequate and inconsistent advice (lack of continuity of care; changeable advice about Covid and pregnancy) • Appreciating good care when it happened • Parents sought information and advice online due to inadequate NHS core provision
**4. First time new parents had diverse feelings with regards to atypical baby social experiences, parent-baby relationships and their child’s development**	• Separation of parents and babies due to hospital restrictions • Concerns over their child’s development due to atypical social experiences and digital technology use
**5. The pandemic affected the working and financial lives of first-time new parents—but sometimes in very different ways**	• Practical and emotional implications of loss of income • Silver linings: early maternity leave and one or both parents working from home • Practical and emotional implications of supply shortages • Paying extra to replace missing services, e.g. bringing partners to private scans
**6. First time new parents had complex and sometimes internally-conflicting thoughts and experiences in relation to digital technology use**	• Parents felt that the remote healthcare appointments were often of poor quality • Use of digital technology seen as a poor replacement for some in-person interactions • Reliance on online information and confusion about contradicting information • Silver linings: feeling appreciative for having digital technology as a resource to create a ‘digital village’ • Silver linings: some families grateful for digital technology as an alternative to in-person health settings for non-urgent care during the pandemic
**7. First-time new parents expressed anger and worry about shifting and contradictory government guidelines during the pandemic**	• Inconsistent and rapidly changing information and guidance about birth processes, Covid risks and Covid vaccines • Feelings of frustration and anger towards individuals in the government breaking guidelines, in contrast to personal sacrifices • First time new parents feeling angry, shocked and saddened in relation to perceived relative de-prioritisation of parents by government guidance
**8. When prompted, first time new parents articulated a range of recommendations for future provision associated with their experiences**	• A space to reflect and talk about experiences • The need to remove restrictions on partners • The need for consistent, accessible information in future • Following up, and checking up on, pandemic first time new parents • The need for social support

## Results

The study’s overall results are visualised in Fig. 2 and described in summary in Table 2. The visualisation in Fig. 2 emphasises the interconnectivity of many of the themes. The results are then discussed in more depth. The more detailed narrative presentation of results has been structured in relation to the eight overarching themes, as summarised in Table 2. Demonstrative excerpts from the data are presented to emphasise the grounding of our findings in the evidence. Each excerpt is followed by some descriptive information about the response and respondent in square brackets. The first number, e.g., ‘144’ refers to the participant number. The letter which follows, e.g. ‘F’ corresponds with the stated gender of the participant. Finally, the date listed, e.g. ‘April 2020’ corresponds with the month and year that the respondent became a (new) parent.

Although some research teams working with rich and plentiful qualitative data may have chosen to focus in greater depth on just a subset of these themes, we have chosen to present all of our findings at a top level in one place. The range and diversity of experiences articulated in the study necessitate presentation within the context of a complex whole. Reading that some new parents felt relief at not having to receive visitors, for example, might easily be misconstrued without the contextualisation that many others experienced the same circumstances as profoundly isolating and lonely.

### First-time new parents’ well-being was impacted

Our data suggest that the well-being of the study’s participants was impacted by the experience of becoming a new parent during the pandemic, but within this theme, diverse and sometimes seemingly contradictory emotions were discussed. Though, in many cases, parents saw the need to follow some, or all, of the guidelines and restrictions imposed during the pandemic, some of these caused distress to new parents and some reported a great impact on their well-being with increased feelings of fear and uncertainty. Encapsulating the significant emotions expressed by many in the dataset, one participant described a sense of ‘sheer terror’ [66, F, July 2020] when her husband had to leave the ward soon after their baby had been born. Some parents also described similar experiences in the period before their baby was born:

*I had to endure the first 15 hours of labour alone without my husband. Partners weren’t allowed in until the woman was moved to the Labour ward. I was alone and scared and felt the time took forever to pass as I had very little to distract me [67, F, April 2020].*

As highlighted in our introduction, rules changed quickly during this period and this rapid change, alongside inconsistent advice, appeared to be an important driver of increased feelings of fear and uncertainty. This sub-theme is particularly exemplified in the following quotation:

*The ever-changing rules, the uncertainty, the fact not even the hospitals had a clue what their own rules were. It was a frightening time as it was, never mind rules changing at the hospital every 5 minutes [144, F, April 2020].*

The guidelines and restrictions left many parents feeling lonely and isolated as they had limited interactions with friends and family. Feelings of loneliness and isolation were experienced throughout the journey to new parenthood, including experiences of appointments pre-birth, the birth itself and social experiences following the birth. Many women described attending ultrasound scans alone or being treated for certain pregnancy-related conditions remotely. The confluence of both was characterised by one mother as contributing to ‘*a very lonely experience all round*’ [106, F, September 2020]. Others emphasised that being isolated at key points leading up to the birth meant they were forced by circumstances to make important decisions on their own:

*It was a lonely experience... It ultimately ended up with me being pressured into making decisions that I didn’t want to make and I had nobody to advocate for me when I needed them [123, F, July 2020].*

Some of the new parents found themselves having mixed feelings about their experience and this seemed to be characterised by an internal emotional dissonance of feeling more than one emotion at the same time. Parents cherished the opportunity to spend time by themselves with their baby, but also sadness at missing out on key experiences, having their partners at appointments, socialising with their friends and family and having other ‘typical’ post-birth experiences. Shifting emotions and internal emotional dissonance during, and after, a period of trauma has been emphasised in past work (e.g., [2]) which highlights that emotional responses to a range of traumatic events can vary in nuanced ways between peritraumatic and posttraumatic time points.

*But in some ways it made me braver and stronger. My emotions and thoughts about the experience change about it regularly. Sometimes I’m grateful for the space and more recently I’m jealous of other pregnant people having support at scans and being able to share their pregnancy with others [27, F, November 2020].*

*I’ll always look back on that period half with sadness and a whole range of things we missed out on and half with gratefulness at the special time I got to spend with my husband and baby [64, F, June 2020].*

In the context of reduced quantity and quality of health care, some parents appeared to struggle in getting the support they needed. Despite systemic inadequacies, many respondents articulated these experiences in terms of their own feelings of shame and inadequacy as new parents, especially with regards to caring for their newborns, getting enough sleep and breastfeeding:

*The fact was he wasn’t gaining weight so I felt there must’ve been something wrong with my milk [...] I didn’t discuss giving up breastfeeding with anyone, I felt ashamed that I’d only managed 3 months but so upset at how traumatic it had been for both of us [36, F, Feb 2020].*

*I was worried about knowing what to do and being able to get rest which I desperately needed. This caused me quite a lot of emotional distress and resulted in me needing to call the midwife during the night in tears as I was so sleep deprived and my baby wouldn't stop crying, I wanted them to take him away and look after him for me because I felt incapable [52, F, Dec 2020].*

Conversely, and reassuringly, parents also articulated feelings of pride associated with having managed to get through a very challenging experience in unprecedented times, taking care of themselves and their newborn babies.

*I am a stronger mother because of it, having done everything with no help [...] for much of the first year it really has made me a lot stronger. When things get tough, I say to myself, ‘I had a baby during the first lockdown, I can do anything [47, F, May 2020].*

### First time new parents felt without the expected ‘village’ but some expressed appreciation for small ‘silver linings’

For most new parents, the period before and after the birth is often filled with communal activities to ease the life transition. In the present study, many respondents emphasised that the experience of being part of a so-called ‘village’ that they had anticipated prior to becoming parents was significantly impacted by the pandemic and the various associated restrictions put in place. Here again, participants shared a diverse range of experiences and emotional responses to those experiences, with some emphasising severely negative impacts and others articulating small ‘silver linings’. The particular circumstances of different families appear to have played an important role in the experiences of parents, for example the timing of their births and the nature of each parents’ work.

With the restrictions on meeting in-person, some participants expressed feelings of grief and disappointment associated with not being able to take part in these activities, including a perceived lack of involvement by their friends, family and colleagues in a momentous life change. Some explained that their transition from working life to new parenthood lacked the important markers of recognition typically associated with this significant life change:

*I had no balloons or banners and no hugs as I left* [183, F, March 2020].

Many wrote about a very unusual experience that became common for many pandemic new parents, that is, not being able to tangibly share the experience of their baby with others:

*Very few of my closest friends and family were able to hold my baby when she was really little and missed the chance to bond that way. Some of my family have yet to meet her due to restrictions and she’s currently 16 months old! [106, F, 2020 Sept].*

New parents often look forward to making friends with other new parents where they could talk about the experience, make sense of their new identity and changes to their life, and share advice. The cancellation of pre-natal classes and mum and baby groups meant many pandemic new parents were unable to create these important bonds.

*I’m grateful that I had a great support group in my NCT group, but again we had never met in person because all our meetings were online. I felt robbed. I still feel robbed. I’m angry that I have 2 years of time that could have been invested in nurturing friendships with women who gave birth at the same time as me, and I haven’t been able to do that. [73, F, May 2020].*

Our analyses again emphasised how different life circumstances played a role in parents’ experiences. The timing of birth relative to various policy changes, and the nature of both parents’ employment, meant experiences differed, as was the case for one mother, who felt she’d missed the ‘window of opportunity’ for building local networks:

*I also had a vision of how my maternity leave would look - attending baby groups and making friends with local mums, which obviously didn't happen. As soon as those groups were able to reopen, I was back at work and I feel that I missed out on making those connections [170, F, 2020 July].*

This lack of an anticipated ‘village’ was clearly experienced and expressed in relation to a lack of physical and emotional support during and immediately after birth itself. As noted above in relation to isolation and loneliness, some mothers were left without someone to help them make important decisions or to advocate for them when they felt that the decisions being suggested by hospital staff were not appropriate*.* For example, one mother expressed significant feelings of anger associated with having been encouraged to have an induction that she later regretted:

*I requested a C-section and was told I couldn't have one. Staff then changed their minds and said it was my right to ask for one but told me only negative things and tried to put me off having one, which worked. I was disassociating by this point, something that was pointed out to me during my birth trauma counselling, and multiple hospital staff failed to recognise this. Had my partner been with me he could have raised this and advocated for me and helped me to understand what was happening [184, F, Sept 2020].*

Many participants experienced distress at having to labour by themselves:

‘*Trauma is my overriding emotion about the birth of my baby […] labouring completely alone* [213, F, June 2020].

Women also had to take care of newborn babies on their own as they recovered from birth, which, for some, had already been very traumatic in itself:

*Because of complications my wife had, it meant she had to stay in for 4 more days after giving birth, yet I was only allowed to be there for 90 mins per day. I could see the effect this was having on my wife as she was essentially bed bound for 4 days with a newborn baby and no real support. As much as the nurses were doing their best they couldn't give her as much support as my wife would have had if either I, her mother or her sister were allowed in as well [194, M, August 2020].*

*My induction was very long and very painful. Five days after I was admitted with minimal visitation from my husband, I finally had a very rough and traumatic labour that ended in an emergency c section. I haemorrhaged, had a uterine rupture, had sepsis and almost died. My husband was shooed out of the hospital and wasn’t allowed back until the following day and only for 2 hours. I was expected to take care of my newborn baby, alone, when I couldn’t even get out of the bed. I was hooked up to so many machines, barely conscious and I was expected to look after my baby alone. I cried and pleaded and cried and pleaded for the staff to let him in, but they wouldn’t. 2 days later I discharged myself, despite being profoundly poorly. My baby was born July 2021, it is worth noting that the government lifted all Covid restrictions the month prior [318, F, July 2021].*

Once released from the hospitals, participants described the need for physical and practical support to take care of their babies and their own well-being. Things they had expected their friends and family would be able to help out with, such as household chores, fell to parents who were in need of physical and mental rest and recovery, due to restrictions on meeting in person. *I was severely sleep deprived and needed someone with me in rooms to listen to the information I was given at these appointments but wasn't allowed it. I gave up trying to breastfeed after 2/3 weeks because I wasn't getting the support I needed because they weren't accessible to me [32, F, May 2020].*

*We went for long periods of time with no one coming into our house [...] all I wanted was for someone to come in and sit with my baby so I could go and have a rest or sleep, or help with a job. I don't think I had that for almost all my maternity leave [122, F, June 2020].*

As noted above, pandemic experiences were diverse. Sometimes the diversity of these experiences itself, along with conflicting opinions, comparisons and misinterpretations of experiences, exacerbated negative social experiences, affecting relationships between new parents, their peers and families. For example, one mother struggled with people telling her that the experience must have had specific benefits, something that deeply conflicted with how she had personally experienced her situation.

*People always said to me that a benefit must have been that there weren't many mums on any of the wards or any families coming in bothering anyone. I found that deeply isolating and lonely [21, F, May 2020].*

Some new parents found themselves in conflict with friends and families, who misunderstood and misinterpreted new parents’ decisions. A commonly misunderstood concern was new parents’ desire to keep their family safe from infection, including their newborns. Choices made in the interests of protecting babies sometimes led to profound conflict with other family members including ‘*driving wedges into our broader family structures*’ [197, F, Oct 2020]. One mother’s account captures the experience particularly well:

*I didn't want any help as I was scared someone might bring in the virus so placed so much pressure on myself and husband. Made it incredibly overwhelming and resulted in relationship issues we are still trying to wade through 8 months later. In laws haven't understood fears and quips are constantly made about not seeing their grandchild and eye rolling when asking to wear a mask. It has broken the relationship to the point I'll be civil for my daughter but that's it [304, F, July 2021].*

Non-birthing parents in the study also struggled with a range of experiences and emotions as a result of being excluded from aspects of the experience of becoming a new parent. Many felt left out and upsettingly ill-equipped to support their partners as hospital restrictions hindered them from being with them during scans and through the complete birth process. Some almost missed the birth of their children as they tried to get into their protective gear before entering the hospitals. Many fathers had to leave soon after the birth of their child.

*Not being allowed into the room for scans was heartbreaking. And the general levels of anxiety around protecting my girlfriend and our bump were huge…[226, M, Sept 2020].*

*My wife was accepted onto the ward and she called me to say I was allowed to join her. I brought in the bags but mistakenly left the bag with food in the car. I wasn't allowed to leave the ward to retrieve it. So my wife had no food and only tap water for the last 24 hours of her labour (we had packed some lucozade) [195, M, August 2020].*

Despite the numerous challenges that the new families were facing, some new parents described silver linings associated with the unique circumstances of becoming a parent during a pandemic. Of these, many foregrounded having more time alone than they had anticipated as holding benefits, particularly to mothers preparing and recovering from birth.

*I enjoyed the distance from some others [...] nobody rubbed my bump or anything like that. I probably received less unsolicited advice than I would have in "normal" times because I saw fewer people [237, F, August 2021]*

*We were able to just figure things out ourselves, and not worry about managing visitors - which was a big positive [187, M, Dec 2020].*

*S*ome families and especially some fathers, had more time to bond with their babies than might otherwise have been the case:

*Due to my work situation I was on furlough for an extended period of time which meant that I had precious extra time to spend with our baby after the birth. [228, M, April 2020].*

It is important to interpret these findings within the broader context of the overall piece. Different circumstances, such as working conditions, played an important role in how pandemic new parenthood was experienced, with other parents in key worker roles having to return to work as planned. At other times, similar circumstances were also experienced in vastly different ways by different new parents.

### Changes to healthcare and related support before, during and after baby arrival impacted new parents’ experiences and well-being

The accounts of new parents in the present study emphasise that pre-, during and post-natal support was reduced and often very different in nature to the care new parents might expect in non-crisis times. New parents’ accounts suggest a feeling that increased concern around the spread of Covid-19 sometimes overshadowed some of the more everyday, but still dangerous, risks associated with childbirth. Some parents described the experiences associated with understaffed wards:

*After going through severe physical trauma in delivering our child she was basically left to fend for herself under disgusting conditions, basically being told to mop up blood and change her bedding herself - even though she could hardly move a muscle. As such she discharged herself the next morning believing I could take better care of her and our new child at home than the hospital could [197, M, October 2020].*

In other cases, diagnoses appear to have been delayed due to an overriding preoccupation with the virus:

*After having my waters broken, I became ill quite quickly, they assumed straight away I had Covid so when I was eventually taken for an emergency c section I was taken to a special Covid theatre. My partner and I, who was only now allowed in to see me, were treated like lepers. When my son arrived, they took him away to isolation on NiCU ward and my partner and isolated on another ward. After two days of not being with my son, I learnt that I had sepsis not Covid, which was far worse. I felt the hospital was so paranoid about Covid that they missed the vital signs of how ill I was [193, F, April 2020].*

New parents in the study were often faced with inadequate and inconsistent information with regards to the care they were to receive and about the guidelines and regulations related to Covid-19. With each NHS Trust developing their own guidelines and restrictions, these factors left some feeling confused, uncertain and, at times, like they had been treated unfairly:

*The lack of care. And inequities in care. If I had given birth in a different postcode, I would have been allowed someone during my induction [223, F, July 2020].*

*My experience of antenatal care was shocking, I was passed around from midwife to midwife, no one knew where I should be going, appointments were missed as they sent me to wrong places, medical things were missed so I ended up needing additional scans when I raised a concern [234, F, January 2021].*

Some parents expressed significant anxieties associated with going into hospital, which was sometimes viewed as placing oneself and one’s baby at risk of getting infected with Covid-19. This was exacerbated by the inconsistency and inadequacy of the information shared during the pandemic, as discussed above. Those who were due to give birth towards the beginning of the pandemic perceived that there was hardly any clear information available or, conversely, too much negative information about Covid-19 circulating and this had exacerbated the already stressful experience of being pregnant.

W*hen Covid hit I became absolutely terrified that I was going to have to go to the one place where Covid was rife - the hospital! My midwife appointments all moved from the local children’s centre to the hospital, and all home births were cancelled. I had no choice but to visit the one place we were all desperate to avoid, and had to do so alone [73, F, May 2020].*

As discussed in more depth in theme 6, many parents sought information and advice online. Face-to-face appointments were reduced, and so too were some of the less formal interactions with friends and family that serve as important sources of information and guidance. In this context, some parents sought this information and guidance using online platforms instead. This included advice about the health of newborn babies, for example:

*There seemed to be limited support from services, no family or friends able to visit and with a colicky baby it was very isolating. Again, I had to rely on online research and Facebook groups for support and advice and I wasn’t always sure how I felt about this [186, F, May 2020].*

It also spanned to how parents prepared for their impending new parenthood and how they decided what to do with their babies to support their development when they arrived:

*I found it difficult having all the antenatal classes cancelled last minute and there being no replacement or alternative to this. I had to rely on my own research to help me prepare for the birth and parenting [186, F May 2020].*

*I have had to do a lot of research online about baby development and activities to do seeing as we were not seen by a health visitor or couldn't go to any classes [78, F April 2020].*

In keeping with a strand of findings focused on ‘silver linings’, participants emphasised their appreciation of good care, when it happened. In some instances, it appears that hospital staff went out of their way to ensure that the new parents got the care that they needed, in spite of staffing shortages.

*[The] Hospital's Labour Ward staff were absolutely incredible and I could not fault them at all. Even with the complications my wife and child experienced they provided my wife with all the support and encouragement she could have asked for and I was made to feel part of the occasion and not just a spare part. Some members of staff even stayed beyond the end of their shift to stay with my wife when things became critical [197, M, October 2020].*

### First-time new parents had diverse feelings with regards to atypical baby social experiences, parent-baby relationships, and their child’s development

Initial baby and mother contact post-birth is argued to be of great importance for both the mother and baby [43]. This is a critical moment that ought to be maintained at all costs unless the baby or mother requires immediate care. For some new parents during the pandemic, this opportunity was not afforded for various reasons, including restrictions specific to individual hospitals. Some parents described feelings of grief associated with these experiences:

*After having my waters broken I became ill quite quickly, they assumed straight away I had Covid [...] When my son arrived they took him away to isolation on NiCU ward and my partner and isolated on another ward. After two days of not being with my son I learnt that I had sepsis not Covid [...] We never got the magical time first time parents get with a newborn [193, F, April 2020].*

The combination of reduced health care before, during and after child-birth, lack of antenatal classes and restrictions on interacting left some new parents feeling they had limited access to information about the appropriate development of their newborn babies. The accounts of parents shared within the study suggest that parent concerns about their babies’ health were not always adequately addressed by health visitors. Clinic appointments were often cancelled or rushed through, and some parents felt that they alone were not adequate for the optimal social development of their babies. The accounts of new parents suggest many compared the development of their babies with what they thought was the ideal. Compared to times of non-crisis, however, they lacked contact with others (professional or non-professional) who might confirm their worries or, conversely, reassure them that they were doing okay. Some concerns related to physical development, and particularly baby weight checks. In other cases, parents worried about their babies’ social development:

*After some time it did begin to concern me about how my baby was being socialised or lack of. I worried about her development and worried that Dad and I weren't enough for her development [...] I worried if not being able to socialise and not witness how "normal" interactions took place and how this might impact her later in life [5, F, September 2019].*

### The pandemic affected the working and financial lives of first-time new parents—but sometimes in very different ways

To try to curb the spread of the virus, the government regulations asked people to work from home. As noted above, different circumstances, such as working conditions, played an important role in how pandemic new parenthood was experienced. For some parents, as noted above, this meant they were able to work completely from home (or be ‘furloughed’) and thus spend more time than anticipated with their newborn babies while earning a normal income. At the same time, many businesses were struggling, and this led to some parents losing their jobs or a reduction in their income, which affected their emotional well-being and their ability to take care of their newborn:

*My fears were also realised when I was made redundant while on maternity leave due to Covid-19. I was furious to discover that I was not the only expectant mother made redundant from the company but felt powerless to question it [7, F, November 2019].*

Other families faced difficult decisions, with new parents or soon-to-be parents ultimately choosing unpaid leave to avoid the risks posed by an unknown virus [24]. In the case of furlough, however, the following account serves as a helpful reminder that similar circumstances were also experienced in vastly different ways by different new parents:

*Due to my work situation I was on furlough for an extended period of time which meant that I had precious extra time to spend with our baby after the birth. Unfortunately, I believe the stress of Covid, my uncertain work situation, family pressures and having a new baby caused me to suffer from depression after the birth. The extra time at home was great but there were periods where I was really just helping out with practical tasks around the house and was not fully present emotionally [228, M, April 2020].*

Something that has perhaps been less explored in existing literature to date is the practical and emotional implications associated with supply shortages, particularly in the early stages of the pandemic. The uncertainty of what the virus was and how long the country would be in lockdown led to panic purchases of supplies such as tissue paper, nappies, baby formula, medication and other supplies. For those who did not have the means to do this, they were left with a low supply of the products that they desperately needed which led to uncertainty and worry over being able to take care of their new family. Many new parents also missed the opportunity to physically go shopping and get ready for the arrival of their baby.

*We couldn’t go shopping for baby things - I had to order a pram online guessing what it would feel like. All the clothes were ordered online but because of the issues with products getting to the UK & staff shortages, there was hardly anything available. I was having to “shop” at 6am when the stock updated on the websites & just grab what I could in newborn sizes, not necessarily to my taste & usually paying more than I wanted [83, F, July 2020].*

*I remember most clearly the worry around emptying shelves and talk of disturbed supply lines. Toilet roll? No chance. Paracetamol for aches and pains and early labour? No chance [19, F, May 2020].*

Another lesser explored dimension of financial impact is the cost to parents who elected to replace missing public services by paying for private ones. The restrictions on partners coming into ultrasound scans was commonly discussed. With some fathers forced to be left out, families who could afford it paid for private scans:

*My partner wasn’t able to experience any of the excitement of seeing his baby move on screen during a scan - we ended up booking and paying for a private one - as this was allowed - so that he could see his baby! [284, F, January 2021].*

As with other broad findings of the study, some parents articulated perceived ‘silver linings’ of the pandemic. The government guidelines to work from home offered some of the new parents more time at home where they could get ready for the baby. This was seen by some as a blessing, with expectant mothers describing extra opportunities to rest pre-birth, not having to worry about being sick while at work and also spending more time with their partners. Some partners also described silver linings, noting that being at home lessened their worries about leaving their partners by themselves in case something happened while they were at work.

*I was able to be with my partner working from home all through the pregnancy, so I guess I didn't have the worry about whether she was okay whilst I was away from the house at work, or what would happen if the baby suddenly arrived [187, M, December 2020].*

### First time new parents had complex and sometimes internally conflicting thoughts and experiences in relation to digital technology use

In an attempt to continue providing care to families while staying safe, the NHS shifted some appointments online. Whilst some valued this intervention, many felt that their remote healthcare appointments were of a poorer quality than they had experienced, or anticipated, their in-person appointments would be. New parents articulated cases of wrong or missed diagnoses and a feeling that appointments were becoming more of a tick box exercise rather than meaningful care.

*Postnatal appointment with a GP at 6 weeks by phone was very poor. I felt unable to say what I really wanted and it felt like a questionnaire and tick box exercise. I didn't get the support I could have early on with my baby's cows milk allergy and reflux [134, F, September 2020].*

Beyond healthcare, the use of digital technology was also seen as a poor replacement for other in-person interactions. Though the online interactions provided a safe way to maintain social interactions with friends and family, many parents said these forms of keeping in touch did not compare to the feeling of having in-person interactions. New parents emphasised physical and practical dimensions, noting that family and friends could not hold their babies or offer practical support to the new families:

*As I left my home country 8 years ago I feel I was used to keeping in touch more over the phone, but the pandemic was challenging even for me. Being pregnant and having a baby is such a special time, our family couldn't help us with chores and baby over the phone and my mum missed the opportunity to hold her newborn granddaughter in her arms. The smell of a newborn cannot be conveyed in a video call [230, F, September 2020].*

Others noted that online provision did not afford some of the freer and more spontaneous dimensions of socialising often taken for granted in the context of in-person parent groups:

*I tried some digital classes like baby massage and post natal mother and baby yoga but I just personally couldn’t enjoy them. I missed the social interaction and found people were quite shy online so there wasn’t the same support and connection made with other mums on this platform [95, F, May 2020]*

As noted in theme 3, many parents sought information, advice and guidance online due to inadequate NHS core provision and conflicting advice. Parents sought information about both medical issues and broader child developmental issues. In addition to expressing dissatisfaction with some of the information they found, parents also described feelings of confusion and anxiety about the contradictory information and advice they encountered online:

*But Mumsnet or Reddit or any other forums really just filled me with anxiety, because there was no answer to any of my questions.... just contradicting advice [4, F, October 2019].*

*I spent a lot of time on forums and Googling, ‘is this normal’ without people to ask. I eventually had to stop as I realised it wasn't any good for my mental health as my anxiety levels were really high [13, F, December 2020].*

*I Googled everything, talked to other mums I've never met about our babies. Asked random women on social media for advice [94, F, September 2020].*

Whilst some parents, including the mother in the latter quote, felt that the support she accessed online was a positive experience given the context, it is clear that many new parents were accessing poor quality sources of information, advice and guidance to inform their decision-making, listing Instagram accounts, Mumsnet, Reddit and peer support groups on Facebook amongst others. As one mother articulated, it is concerning that parents appear to be accessing information that is of poor quality and, further, that the contradictory nature of this information itself is a negative influence on new parental well-being:

*There is so much conflicting information out there, and I believe a great deal of it is toxic and actually I believe it is dangerous and detrimental to many parenting journeys [41, F, December 2020].*

As others have noted (e.g., [31]), further work is needed to interrogate how new parents find and critically evaluate sources of information, advice and guidance online, both within and beyond times of global crisis.

In common with other findings of the study, some parents emphasised ‘silver linings’ associated with digital technology. Though most were disappointed that digital support had replaced expected in-person experiences, digital technology nonetheless afforded new parents opportunities to continue interacting with their family, friends and other new parents. Though online engagement could not meet practical support needs, it provided opportunities to share experiences with loved ones and make new friends who were in a similar situation.

*I signed up for a NCT group which ended up being virtual due to the pandemic. I'm so glad I did it though as we're in regular contact. When we couldn't meet in person we set up virtual coffee morning and afternoon chats so we could keep in touch. This was a real life line and was very important to me [14, F, May 2020].*

*Digital technology was so important for things like FaceTime as that was the only way relatives could see him, it would have felt worse if we did not have these [33, F, November 2020].*

In addition to the social support that digital technology enabled, new parents also viewed it as a good alternative to non-critical health care and appointments. Parents who resumed work also saw this as a blessing as they could attend the virtual appointments instead of driving to the hospitals.

*I would like more things to stay online because as a working mum, getting to things face to face is sometimes more challenging [306, F, June 2021].*

### First-time new parents expressed anger and worry about shifting and contradictory government guidelines during the pandemic

For some of the new parents, the experience of being a first-time parent was dominated by inconsistent and rapidly changing information and guidance about birth processes, Covid-19 risks and vaccines. Many felt that clearer and more consistent information and guidance would have reduced anxiety and uncertainty.

*For most of the pregnancy, I felt alone & terrified about what this virus could do to my baby. One day pregnant women were told not to leave their houses at all because it’s so dangerous then the next day, we were told it was fine but there was no evidence to back anything up [83, F, July 2020].*

*The main negative I found for me personally was the anxiety of trying not to contract Covid amongst the incredible clash of mixed messages regarding vaccination during pregnancy. In the beginning it was a big no-no. Then the advice was “read up about it and talk to your doctor and midwife”, however all the doctors and midwives I had flat out refused to speak to me or advise me about it which didn’t give me confidence in having it during pregnancy. Then towards the end there was a huge push for it which felt overwhelming considering the mixed messages [255, F, September 2021].*

At a time when many were struggling with following the strict restrictions imposed by the UK government at the time, many new parents expressed anger when it was revealed that some officials in the UK Conservative government were having parties during the lockdowns whilst the UK population were making do with having minimals interactions with others and experiencing restrictions with healthcare.

*I'm glad someone is doing this research as I don't think people understand the negative impacts of Covid on parents and families. The recent revelations about Downing Street parties has really really upset me. We stuck to all the rules, had a really difficult time and again, it just seems like many people don't realise how hard it is to become a parent in these circumstances [163, F, May 2020].*

In a similar vein, many new parents felt baffled, angry and deprioritised in relation to official rules, policies and guidance. At points, members of the public could freely interact in bars, coffee shops and other social spaces, whilst women were denied crucial social support, both in hospitals and following their return home.

*I find it appalling that there was a time where people were not allowed to sit down outdoors - I remember being scared that a neighbour would call the police when I sat down on the grass momentarily on my first post-c section walk. A friend was actually moved on by police for breastfeeding her newborn on a park bench [18, F, April 2020].*

*My daughter was transferred to [A] Hospital. There was a ridiculous rule in place where my husband was allowed everywhere in the hospital with me, except our baby's room. Only one person was allowed in the room at one time. We could have a coffee in Starbucks downstairs, we could walk around together, we even chatted on the hallway when swapping over to see our daughter. But we couldn’t actually be in that one room together. The one room where we both needed to be, together. It made a horrific situation almost unbearable [156, F, August 2020].*

*At that point in time the rules said that you could meet 6 people in a pub. My husband had been in the hospital for 6 hours, only 1 floor up from the recovery ward - why separate him from me when I was at my most vulnerable and anxious? [219, F, October 2020].*

### When prompted, first time new parents articulated a range of recommendations for future provision associated with their experiences

Many parents commented that sharing their experiences via the survey gave them a much-needed chance to reflect and ‘talk’ about their experiences.

*I've felt liberated as a Dad to be able to speak about our experiences and my interpretations of them. Dads don't always get a fair chance to just talk about things and I think it's vitally important that this survey is being run. [197, M, October 2020]. *

*I like to share my experience as I still feel I have not had the chance to recover from becoming a parent in lockdown. Talking about it makes me feel someone is hopefully listening! [214, F, April 2020].*

*Thank you for running the study…This has been the most wonderfully validating way to recount everything I went through. It's the first time I've written it down, and read it back. Wow, I went through a lot, I achieved a lot, and I should be proud of myself. I think the voices of new parents were something completely negated in the pandemic restrictions imposed on society. Of course, safeguarding was important, but I cannot even bear the thought of those women who had to go into their scans alone, receive the worst news imaginable, alone, and then have to go outside and deliver that news to their partner.; It's cruel [103, F, July 2020].*

Survey responses foregrounded a sense that pandemic new parents had been granted very few opportunities to share and process their unusual and traumatic experiences, or to see their experiences reflected back in media coverage. Many were thankful that they got the chance to speak about their experiences through taking part in the survey, and appeared to find this therapeutic and felt it had helped them to process and recover from these experiences.

Though many respondents were understanding of the need to make some personal sacrifices in the interests of stopping the spread of the virus, the access restrictions applied to non-birthing parents pre-, during and post birth appear, understandably, to have caused particular distress. Mothers were left alone at one of the most vulnerable times, whilst non-birthing parents were excluded from large parts of the process. Removal of these restrictions was therefore one of the main recommendations, expressed by many of the new parents taking part.

*I truly hope if something like this were to happen again, birth partners are not classed as visitors. It’s barbaric that women had to go through so much trauma alone when I personally do not think the benefit of exclusion outweighed the risk. That is the only thing that would be helpful and useful [207, F, November 2020].*

Parents also emphasised the need for consistent, accessible information during a time of uncertainty:

*Streamlined advice from trusted resources that is evidence based rather than idealistic and based on opinions [309, F, June 2021].*

Many respondents felt a sense of abandonment due to the limited health care and mental health support that they received during the pandemic. Many felt strongly that not enough had been done to check up on how pandemic new parents were getting on:

*More focus on postnatal follow up, but in particular the mothers mental health. I am hearing from other mothers that in some of their 8 week check ups they have been told it is just to focus on the baby and not them. This is dangerous and detrimental [213, F, June 2020].*

Indeed, others went further, suggesting that there would be a continued need to follow-up on the new parents and babies after the end of the pandemic, with several characterising themselves and their peers (or their children and their peers) as a unique or forgotten generation:

*For it to actually be recognised that we missed out on so much and a lot of people had a very difficult birth experience. I think the wrong call was made not to allow health visitors to visit. It would be nice to think that our children will not be the ‘forgotten generation’ of ‘lockdown babies’ that missed out [229, F, April 2020].*

*Becoming a new parent during a national lockdown was an extremely challenging time. I am not aware of any additional support that has been offered to those who became new parents during the covid national lockdowns and this is something which needs to be recognised and for support to be put in place. It isn't too late [137, F, November 2020].*

In a similar vein, experiences of pandemic new parenthood were diverse, and some parents felt that there was a misunderstanding of their experience by others. In this context, many emphasised the need for parents to have a form of support from others in a similar position, particularly those with shared experiences.

*If you're allowed to be in the same room with women going through exactly the same thing as you, it makes a world of difference. The sense of isolation and/or loneliness is one of the hardest aspects to deal with and getting out of the house and seeing other people can help you forget about the insanity for a little while [177, F, October 2020].*

## Discussion

Becoming a new parent in ordinary times brings with it changes in emotions, mental and physical health, as well as family dynamics and identity. The Covid-19 pandemic brought drastic changes in policy and practice that impacted new parents. We aimed to understand the impact of these changes on new parents, and in particular, how they shaped the experiences of the journey to becoming a first-time parent. We conducted a qualitative survey online to provide new parents across the UK the opportunity to describe their experiences of pregnancy, birth and through to the postnatal period. Many respondents found the opportunity to share their experience helpful and were grateful for the research study being carried out, having felt ‘forgotten’ during the pandemic. Drawing on considerable coding, discussion, drafting and redrafting, we developed eight overarching themes from the data.

Our first research question was how the pandemic had impacted new parents’ wellbeing. A clear and overarching theme was impact on first-time parents’ well-being. Negative impacts on new parents’ emotional well-being were clearly evidenced, with changes in healthcare provision and practices particularly affecting well-being. New parents foregrounded a range of causes for these negative impacts, including: a sense of deep isolation at being left without the anticipated ‘village’; fears of themselves or their babies falling ill; uncertainty and inconsistency in rules affecting their care; reduced healthcare support; maternity policies limiting partners being there for key moments; loss of income for some families; and supply shortages. In regard to emotional well-being, many parents reported feeling fear, loneliness, isolation, and uncertainty. In addition to these feelings, a sense of emotional dissonance was apparent as new parents grappled with trying to understand a range of complex opposing emotions. Mixed emotions, or feelings of ambivalence have been associated with poor well-being in parents [32]. However, they can be a common feature of new parenthood, for example new fathers have reported feeling both ‘joy and trouble’ [13]. The conditions of the pandemic appeared to exacerbate these mixed feelings, as families were thrust into life as a small family unit isolated from their extended social networks. Feelings of relief at delivering babies safely and being able to bond were often coupled with a deep sense of isolation and loneliness. Many parents talked about a sense of pride at coping during a difficult time, combined with feelings of fear and uncertainty about what the future might hold. These mixed feelings may have impacted new parents' well-being as they struggled to make sense of opposing emotions.

Our second research question was how the pandemic had impacted parents' social support. The findings highlight how critical social support is for new parents. Without the anticipated and expected social support during the pandemic, many parents appear to have been left vulnerable. Recent quantitative research has demonstrated associations between social support and mental health in women during the pandemic both in pregnancy [9] and post-partum [39]. In our study, many new parents described feeling lonely and isolated and with reduced practical and emotional support. It is noteworthy that groups for first-time new parents have been considered as important as other forms of postnatal service provision because of the importance of meeting others experiencing such a life transition [15]. Social support may have a direct effect on a person’s sense of well-being, or alternatively can act as a protective factor in buffering stress levels [8].

Our third research question sought to understand the role digital technology played in first-time parents during the pandemic. As in-person contact was reduced during the pandemic, much of the world shifted online. The study’s participants foregrounded complex and sometimes internally conflicting feelings about their engagement with digital technologies during the pandemic. Some felt relieved to have it as a way to connect with family and friends and build a ‘digital village.’ Some parents felt it did not replace in-person interactions and in addition, some felt pressure to constantly keep family and friends updated. With regards to healthcare, many parents felt remote appointments were poor quality whilst others felt they were a good alternative, particularly for non-urgent appointments and as a way to avoid going to the hospital and risking catching the virus. Parents also reported using digital technology as a way to get information and advice when advice from professionals was either lacking or inconsistent. The informational gap left behind by professionals during this time, and the clear use by parents of poor-quality online sources is a concern. The study indicates the need for first time new parents to receive both advice on where to find evidence-based information and guidance in developing critical digital literacy to make sound decisions about finding, evaluating and acting on information they find online, including on social media [42] Our fourth research question focused on parents' concerns for their children during the pandemic. Indeed, in our study, many parents reported feeling worried about the impact of atypical social experiences on their child’s development. These concerns may have been exacerbated by media coverage during the pandemic, where a common narrative developed around ‘lockdown babies’ falling behind which may have fed into parents feeling anxious about the impact of lockdowns on their child’s development (e.g., see USA Today [41] for an example). This contrasts with academic studies concluding that many infants and toddlers gained more vocabulary than was to be expected during this time [23]. Positive outcomes were reported particularly in cases where parents were high in self-efficacy and social support [45]. Professionals have a vital role to play in providing parents with much needed evidence-based information to support their new role as parent and alleviate worries.

Among the clear impacts this time had on new parents' well-being, silver linings cut across many of the eight themes. Participants reported appreciating time as a family unit to prepare for impending parenthood, time to recover and bond as a new family, feelings of pride related to coping during this difficult time, and valuing having early maternity leave and often time with their partner at home. The idea of ‘blessings and curses’ is a theme echoed in other qualitative studies of parents during the pandemic (e.g., Joy et als’ work on antenatal period., 2020). Many families over the UK have reported benefiting from spending more time together as a family; fathers in particular spent more time with their children in 2020 enabling them to build strong bonds [4]. Furthermore, the results emphasised how policy and practice have a huge effect on how parents experience becoming a parent. In particular, parents were keen to discuss their experiences of good care when it happened. Good care may act as an important buffer for the effects of other stressors on outcomes and well-being [28]. It is clear maternity units were stretched during the pandemic. New parents felt keen to show their appreciation for good care during their birth, and in particular to the midwives and doctors who went above and beyond to bring their new baby into the world safely at a time when their own safety was at risk. It was interesting to see how different parents interpreted distressing things happening to them during the birth but how the impact on well-being was buffered, in some cases, when the parent had experienced good and compassionate care from health professionals.

Also cutting across the data, however, was a clear picture that the Covid-19 pandemic impacted on first time new parents differently. Other studies have highlighted that established disparities in experiences of pregnancy and new parenthood were exacerbated during the pandemic [20]. Our own study was not designed to measure sociodemographic disparities, but nonetheless foregrounded that a range of particular circumstances played important roles in shaping the experiences of different pandemic new parents. Experiences were quite different for parents giving birth at different time points during the pandemic, as they were clearly subject to quite different levels of restriction. The nature of parents’ work played an important role in experiences, with some, including furloughed staff and those able to work from home highlighting positive impacts, whilst others who were key workers or found themselves out of work experiencing negative impacts. Finally, it is clear that even when individuals within the study were subject to quite similar circumstances, their individual experiences of them could be quite diverse. For example, some parents, who were extremely anxious about Covid risks, felt relief at stringent Covid control measures in hospitals, whilst many others experienced the same measures with horror.

We acknowledge limitations of the study, which was carried out during a time of national crisis and was, like all studies, methodologically imperfect. Firstly, we did not collect extensive demographic information on parents (such as their age) or biomedical aspects of the birth or whether babies required aftercare. This was still indicated in some accounts, but not all. This limits our ability to ascertain how representative our sample is to a broader population along these background characteristics. Secondly, gathering qualitative data using a variety of methods including interviews or focus groups would have likely supported generation of a richer qualitative data set. Researchers globally were limited legally and ethically by Covid restrictions on in-person research. Whilst online interviews would have been a useful addition, we consider an open-response survey the right choice for the context. The survey was anonymous at-source, and participants were able to share as much or as little as they wanted in an open-ended format. The survey allowed us to gather a greater number and range of responses, as well as reducing the time and organisational burden on participants. Thirdly, the participant sample was less diverse than we had hoped with regards to gender and ethnicity. Given that the present study highlights significant differences in the experiences of new parents, it is all the more important that future work in future crisis and non-crisis situations examines the roles that a range of factors including sex and gender, race and ethnicity and socioeconomic status play in new parents’ experiences. Nevertheless, diverse experiences were shared in terms of the impact of policies and practice on well-being (from highly negative experiences to reporting silver linings) and seeing digital technology as a burden, poor replacement or a saviour. Our study was unique in being able to identify themes from the journey to new parenthood during a time of national crisis. Unlike many studies, we did not just focus on a single time period, but three key stages along the way to becoming a new parent. This allows us to get a holistic picture of the experience during key stages of the journey. We discuss below the conclusions and recommendations we can make based on these findings.

## Conclusions and recommendations

Our study has given new parents during the pandemic an opportunity to share and reflect on their experiences and provided a much-needed context to elevated levels of poor mental health in parents who had children during the pandemic. The results highlight the importance of high quality in-person care, social support and reliable information in supporting well-being during this major life transition. Drawing on our findings, we propose five recommendations that apply to provision for new parents going forward, both within and beyond possible future periods of national crisis.

Firstly, there is a need for broader awareness and recognition of new parenthood as a major life transition, both in general and in particular in times of crisis such as the Covid-19 pandemic. Many parents told us they felt forgotten and left behind, and new parents clearly need more opportunities for meaningful involvement in the policies and practices that affect them. Secondly, the results highlighted the importance of social support before, during and after childbirth. Pandemic new parents very clearly consider the exclusion of non-birthing parents from large parts of the birth process to have been a step too far, particularly when risks in both directions are weighed. It is also clear that many new parents rely on networks of new parents to share experiences and advice. More investment in local parent groups, available to all free of fees, would act as a huge support for new parent’s well-being during this major life transition. Thirdly, parents valued good in-person care, but changes to online provision fell short. Perhaps even more concerningly, many services in the UK have still not resumed to pre-pandemic levels, including provision of face-to-face GP appointments, health checks for new mothers and baby weigh in clinics. We strongly stress the importance of this in person provision for new parents in feeling supported, reassured, and getting evidence-based advice. Fourthly, the silver linings reported by some mothers and fathers highlight the benefits of being at home during the final stages of pregnancy and the value in having a partner around at home to take an active role in parenthood and offer the birthing partner support and care It is noteworthy that for some families, during this time of great upheaval and reduced provision in other areas, this was a real strength to hang on to. Finally, the significant negative impacts of shifts in both policy and the advice provided to parents emphasises the importance of clear, evidence-based and broadly consistent advice for new parents. Whilst we acknowledge that policy and parent advice during the pandemic had to be guided by the limited and emergent available evidence, a lack of clarity in the advice given, rapid reversals in guidance and an overall lack of logical coherence across national policies contributed to feelings of confusion, distrust, and de-prioritisation amongst new parents. The results also suggest parents are often little prepared for the social and psychological changes that happen during this life transition and it would be beneficial for antenatal groups—as well as covering practical aspects of becoming a parent—to cover wider issues including changes in identity, social networks and the importance of honest discussions with family on boundaries and plans for the postnatal period. If we can take something away from the accounts of new parents during this unique window of time, it is that care and support for new parents should not be deprioritised during times of crisis.

## Supplementary Information

Supplementary Material 1.

## Data Availability

The parent survey questions can be found in the Supplementary Materials. A summary, including quotes from anonymised parent accounts, can be found on our study website here (https://sites.google.com/sheffield.ac.uk/pandemicparenting/home/). After publication, we will provide a full summary of the findings with more accounts on our study website and the full anonymised datasets of parent accounts is available from the corresponding author on reasonable request.
