# Supplementary material for: Becoming a new parent during the pandemic: experiences of pregnancy, birth, and the postnatal period
Source: BMC Pregnancy Childbirth. 2025 Jan 17;25:39. doi: 10.1186/s12884-024-07110-w (PMC11742233; doi:10.1186/s12884-024-07110-w)
Supplement: Supplementary file 1 — Supplementary Material 1. [file 12884_2024_7110_MOESM1_ESM.docx]

**Supplementary Materials 1.** A list of the questions in the parent survey.

*Your Experiences*

We will ask for you to share your experiences before, during and after the arrival of your baby. We are interested in your experience in your own words. Feel free to share your experiences in all three of these areas or just the ones that stand out for you the most.

Thinking about your pregnancy (or the time before your baby arrived) and the Covid-19 pandemic, what stands out for you about the experience? We're interested in your own unique perspective, which might include positives and negatives.

[Open text response]

Thinking about the birth of your baby (or the process of your baby arriving) and the Covid-19 pandemic, what stands out for you about the experience? We're interested in your own unique perspective, which might include positives and negatives.

[Open text response]

Thinking about the time after your baby or child arrived during the Covid-19 pandemic, what stands out for you about the experience? We're interested in your own unique perspective, which might include positives and negatives.

[Open text response]

Do you feel digital technology has played a particular role (or roles) in your experiences of becoming a new parent during the pandemic? We’re interested in any experiences you’d like to share - both good and bad.

[Open text response]

What do you think would be helpful for other new families who might go through a similar experience as you?

[Open text response]

What year did your baby arrive?

2019

2020

2021

2022

What month did your baby arrive?

January

February

March

April

May

June

July

August

September

October

November

December

We are interested in how parents have found it taking part in our study. Can you tell us anything about how reflecting on your experiences has made you feel? Or what you think about the study we are running?

[Open text response]

*Information about you and your family*

Some studies have suggested that families in certain areas with tighter restrictions, those of certain ethnicities, or those with less resources may have faced unique challenges during the pandemic. So that we can have a better understanding of how representative our sample is, can you give us the following information?

What continent do you live on?

Africa

Asia

Europe

Oceania

North America

South America

What country do you live in?

[Open text response]

How would you best describe your ethnicity?

White - British

White - Another background

Black - African

Black - British

Black - Caribbean

Black - Another background

Asian - Indian

Asian - Pakistani

Asian - Bangladeshi

Asian - Another background

Chinese

Hispanic or latino

Mixed ethnicity

Another background

What term best describes the relationship with your child?

Biological parent

Adoptive parent

Foster parent

What term best describes your gender?

Female

Male

Non-binary

Transgender

Intersex

Something else

I prefer not to say

If you consider yourself as having a disability, if you want to, please give us more information here.

[Open text response]
